# Supplementary material for: Rotating Night Shift Work and Risk of Incident Cardiovascular Disease and Mortality by Chronotype: A Prospective Cohort Study in Nurses’ Health Study II
Source: medRxiv. 2026 Sep 17:2026.09.16.26363233. Preprint. [Version 1] doi: 10.64898/2026.09.16.26363233 (PMC13596606; doi:10.64898/2026.09.16.26363233)
Supplement: Supplementary file 1 [file NIHPP2026.09.16.26363233v1-supplement-1.pdf]

medRxiv preprint doi: https://doi.org/10.64898/2026.09.16.26363233; this version posted September 17, 2026. The copyright holder for this preprint (which was not certified by peer review) is the author/funder, who has granted medRxiv a license to display the preprint in perpetuity.

It is made available under a CC-BY 4.0 International license .

Supplemental Table 1. Prospective associations of alternative rotating night shift work exposures with risk of cardiovascular disease, N = 69,458

			Hazard Ratio (95% CI)		
	Cases / Person-years	Crude incidence per 100,000 PY	Age-adjusted model	Model 1*	Model 2†
Duration of rotating night shift work (4 categories)					
- None	265 / 217194	122	Ref.	Ref.	Ref.
- <5 years	459 / 394657	116	1.00 (0.86, 1.16)	0.98 (0.84, 1.14)	0.95 (0.81, 1.10)
- ≥5 to <10 years	166 / 114023	146	**1.31 (1.08, 1.60)**	**1.24 (1.02, 1.51)**	1.11 (0.91, 1.35)
- ≥10 years	115 / 74757	154	**1.26 (1.01, 1.57)**	1.16 (0.93, 1.46)	1.00 (0.80, 1.26)
Duration of rotating night shift work (complete case only: n = 22,794) ‡					
- None	89 / 75960	117	Ref.	Ref.	Ref.
- <5 years	137 / 132115	104	0.91 (0.70, 1.19)	0.90 (0.69, 1.18)	0.85 (0.65, 1.11)
- ≥5 years	83 / 57732	144	1.33 (0.98, 1.80)	1.26 (0.93, 1.71)	1.09 (0.80, 1.49)
Per 5-year increase	309 / 265807	116	**1.13 (1.00, 1.27)**	1.11 (0.98, 1.25)	1.05 (0.93, 1.19)
P Value for Trend	NA	NA	**0.0423**	0.1008	0.4114
Ever shift work					
- Yes	740 / 583437	127	1.09 (0.95, 1.26)	1.05 (0.91, 1.21)	0.98 (0.85, 1.14)
- No	265 / 217194	122	Ref.	Ref.	Ref.
Time since quitting rotating night shift work §					
- Current	77 / 51332	150	Ref.	Ref.	Ref.
- <12 years	159 / 112907	141	0.93 (0.71, 1.22)	0.95 (0.72, 1.25)	0.96 (0.73, 1.27)
- 12 to 24 years	209 / 167404	125	0.83 (0.64, 1.08)	0.87 (0.66, 1.15)	0.92 (0.69, 1.21)
- >24 years	295 / 251795	117	**0.68 (0.53, 0.88)**	0.77 (0.58, 1.02)	0.88 (0.66, 1.17)
Per 5-year increase §	740 / 583437	127	**0.95 (0.92, 0.97)**	**0.96 (0.93, 0.99)**	0.98 (0.95, 1.01)
P Value for Trend §	NA	NA	<.0001	0.0096	0.1911
Shift work status					
- Never shift worked	265/ 217194	122	Ref.	Ref.	Ref.
- Current	77 / 51332	150	**1.40 (1.08, 1.80)**	1.30 (1.00, 1.70)	1.12 (0.86, 1.46)
- Past shift work	663 / 532105	125	1.07 (0.92, 1.23)	1.03 (0.90, 1.19)	0.97 (0.84, 1.12)
Night shift work frequency in 2009 (n = 65,278)					
- None	772 / 650522	119	Ref.	Ref.	Ref.
- 1-6 nights per month	84 / 54824	153	**1.38 (1.10, 1.73)**	**1.31 (1.04, 1.65)**	1.25 (0.99, 1.57)
- ≥7 nights/month	67 / 47943	140	1.20 (0.94, 1.55)	1.15 (0.88, 1.48)	0.97 (0.75, 1.26)
Per 5 nights/month increase	923 / 753289	123	1.07 (0.99, 1.16)	1.05 (0.97, 1.14)	0.99 (0.91, 1.08)
P Value for Trend	NA	NA	0.0843	0.2628	0.7991

* Model 1: Age, ethnic background, census-tract median family income, marital status, workplace, family history of MI and/or stroke, menopausal status, and postmenopausal hormone therapy.
† Model 2: Model 1 + history of diabetes/hypertension/hypercholesterolemia, aspirin use, smoking status, sleep duration, physical activity, body mass index (BMI), Alternate Healthy Eating Index-2010 score (AHEI-2010), and alcohol use. Census-tract median family income, AHEI-2010 score, BMI, physical activity, and alcohol use were modeled as continuous variables.
‡ This shift work variable was assessed through the 2007 questionnaire cycle and treated as a fixed (non–time-dependent) variable. Participants with missing data on shift work duration in 2007 or any earlier cycle were excluded from the analysis. All other shift work variables were time-dependent, assessed at baseline in 2009 and subsequently updated in 2011 and 2013.
§ Among current and former shift workers (n = 50,737). NA: Not Applicable

medRxiv preprint doi: https://doi.org/10.64898/2026.09.16.26363233; this version posted September 17, 2026. The copyright holder for this preprint (which was not certified by peer review) is the author/funder, who has granted medRxiv a license to display the preprint in perpetuity.

It is made available under a CC-BY 4.0 International license .

Supplemental Table 2. Prospective associations of alternative rotating night shift work exposures with all-cause mortality, N = 69,458

			Hazard Ratio (95% CI)		
	Cases / Person-years	Crude incidence per 100,000 PY	Age-adjusted model	Model 1*	Model 2†
**Duration of rotating night shift work (4 categories)**					
- None	406 / 218577	186	Ref.	Ref.	Ref.
- <5 years	863 / 397144	217	**1.23 (1.09, 1.38)**	**1.20 (1.07, 1.36)**	**1.17 (1.04, 1.32)**
- 5-10 years	277 / 114979	241	**1.44 (1.23, 1.67)**	**1.36 (1.17, 1.59)**	**1.29 (1.10, 1.50)**
- ≥10 years	231 / 75343	307	**1.66 (1.41, 1.95)**	**1.51 (1.28, 1.77)**	**1.40 (1.18, 1.65)**
**Duration of rotating night shift work (complete case only: n = 22,794) ‡**					
- None	122 / 76424	160	Ref.	Ref.	Ref.
- <5 years	247 / 132821	186	1.21 (0.97, 1.50)	1.19 (0.95, 1.48)	1.17 (0.94, 1.46)
- ≥5 years	132 / 58206	227	**1.48 (1.15, 1.91)**	**1.38 (1.07, 1.78)**	**1.32 (1.03, 1.71)**
Per 5-year increase	501 / 267452	187	**1.20 (1.11, 1.31)**	**1.18 (1.08, 1.28)**	**1.17 (1.07, 1.27)**
P Value for Trend	NA	NA	**<.0001**	**0.0003**	**0.0007**
**Ever shift work**					
- Yes	1371 / 587466	233	**1.32 (1.18, 1.48)**	**1.27 (1.14, 1.43)**	**1.22 (1.09, 1.37)**
- No	406 / 218577	186	Ref.	Ref.	Ref.
**Time since quitting rotating night shift work §**					
- Current	104 / 51732	201	Ref.	Ref.	Ref.
- <12 years	290 / 113677	255	1.16 (0.92, 1.45)	1.20 (0.95, 1.51)	1.19 (0.95, 1.50)
- 12 to 24 years	393 / 168625	233	1.01 (0.81, 1.25)	1.08 (0.86, 1.36)	1.10 (0.87, 1.38)
- >24 years	584 / 253433	230	0.90 (0.73, 1.11)	1.03 (0.82, 1.30)	1.11 (0.88, 1.40)
Per 5-year increase §	1371 / 587466	233	**0.97 (0.95, 0.99)**	0.99 (0.97, 1.01)	1.00 (0.98, 1.02)
P Value for Trend §	NA	NA	**0.0016**	0.2837	0.9720
**Shift work status**					
- Never shift worked	406 / 218577	186	Ref.	Ref.	Ref.
- Current	104 / 51732	201	**1.36 (1.09, 1.69)**	**1.31 (1.05, 1.64)**	1.21 (0.97, 1.51)
- Past shift work	1267 / 535735	237	**1.32 (1.18, 1.48)**	**1.27 (1.14, 1.42)**	**1.22 (1.09, 1.37)**
**Night shift work frequency in 2009 (n = 65,278)**					
- None	1343 / 654714	205	Ref.	Ref.	Ref.
- 1-6 nights per month	111 / 55250	201	1.02 (0.84, 1.23)	1.02 (0.84, 1.24)	1.00 (0.82, 1.21)
- ≥7 nights/month	135 / 48301	280	**1.39 (1.17, 1.66)**	**1.34 (1.12, 1.61)**	**1.22 (1.02, 1.47)**
Per 5 nights/month increase	1589 / 758265	210	**1.12 (1.06, 1.19)**	**1.10 (1.04, 1.17)**	**1.06 (1.00, 1.13)**
P Value for Trend	NA	NA	**0.0001**	**0.0016**	**0.0465**

* Model 1: Age, ethnic background, census-tract median family income, marital status, workplace, family history of MI and/or stroke, menopausal status, and postmenopausal hormone therapy.

† Model 2: Model 1 + history of diabetes/hypertension/hypercholesterolemia, aspirin use, smoking status, sleep duration, physical activity, body mass index (BMI), Alternate Healthy Eating Index-2010 score (AHEI-2010), and alcohol use. Census-tract median family income, AHEI-2010 score, BMI, physical activity, and alcohol use were modeled as continuous variables.

‡ This shift work variable was assessed through the 2007 questionnaire cycle and treated as a fixed (non–time-dependent) variable. Participants with missing data on shift work duration in 2007 or any earlier cycle were excluded from the analysis. All other shift work variables were time-dependent, assessed at baseline in 2009 and subsequently updated in 2011 and 2013.

§ Among current and former shift workers (n = 50,738). NA: Not Applicable

medRxiv preprint doi: https://doi.org/10.64898/2026.09.16.26363233; this version posted September 17, 2026. The copyright holder for this preprint (which was not certified by peer review) is the author/funder, who has granted medRxiv a license to display the preprint in perpetuity.

It is made available under a CC-BY 4.0 International license .

Supplemental Table 3. Prospective association between chronotype, rotating night shift work, and myocardial infarction, NHSII (N = 69,458)

			Hazard Ratio (95% CI)		
	Cases / Person-years	Crude incidence per 100,000 PY	Age-adjusted model	Model 1*	Model 2†
**Duration of rotating night shift work**					
- None	170 / 217317	78	Ref.	Ref.	Ref.
- <5 years	300 / 394840	76	1.02 (0.84, 1.23)	0.99 (0.82, 1.20)	0.95 (0.78, 1.15)
- ≥5 years	184 / 188895	97	**1.32 (1.07, 1.63)**	1.22 (0.98, 1.51)	1.05 (0.85, 1.30)
Per 5-year increase	654 / 801052	82	**1.11 (1.03, 1.19)**	1.07 (1.00, 1.16)	1.02 (0.94, 1.10)
P Value for Trend	NA	NA	**0.0060**	0.0647	0.6369
**Chronotype**					
- Definite morning	207 / 268866	77	0.95 (0.80, 1.13)	0.97 (0.81, 1.15)	1.06 (0.89, 1.26)
- Intermediate (neither and more)	340 / 435257	78	Ref.	Ref.	Ref.
- Definite evening	107 / 96928	110	**1.40 (1.12, 1.74)**	**1.33 (1.07, 1.65)**	1.11 (0.89, 1.39)
Per 1-level increase toward evening	654 / 801052	82	**1.18 (1.05, 1.33)**	**1.14 (1.02, 1.29)**	1.01 (0.90, 1.14)
P Value for Trend	NA	NA	**0.0061**	**0.0258**	0.8850

* Model 1: Age, ethnic background, census-tract median family income, marital status, workplace, family history of MI and/or stroke, menopausal status, and postmenopausal hormone therapy.

† Model 2: Model 1 + history of diabetes/hypertension/hypercholesterolemia, aspirin use, smoking status, sleep duration, physical activity, body mass index (BMI), Alternate Healthy Eating Index-2010 score (AHEI-2010), and alcohol use. Census-tract median family income, AHEI-2010 score, BMI, physical activity, and alcohol use were modeled as continuous variables.

NA: Not Applicable

medRxiv preprint doi: https://doi.org/10.64898/2026.09.16.26363233; this version posted September 17, 2026. The copyright holder for this preprint (which was not certified by peer review) is the author/funder, who has granted medRxiv a license to display the preprint in perpetuity.

It is made available under a CC-BY 4.0 International license .

Supplemental Table 4. Prospective association between chronotype, rotating night shift work, and stroke, NHSII (N = 69,458)

			Hazard Ratio (95% CI)		
	Cases / Person-years	Crude incidence per 100,000 PY	Age-adjusted model	Model 1*	Model 2†
Duration of rotating night shift work					
- None	96 / 217332	44	Ref.	Ref.	Ref.
- <5 years	160 / 394881	41	0.96 (0.75, 1.24)	0.95 (0.74, 1.23)	0.93 (0.72, 1.20)
- ≥5 years	98 / 188929	52	1.24 (0.94, 1.65)	1.20 (0.90, 1.60)	1.08 (0.81, 1.45)
Per 5-year increase	354 / 801141	44	1.09 (0.99, 1.21)	1.08 (0.97, 1.19)	1.04 (0.94, 1.15)
P Value for Trend	NA	NA	0.0759	0.1470	0.4602
Chronotype					
- Definite morning	100 / 268920	37	**0.75 (0.59, 0.96)**	**0.76 (0.60, 0.97)**	0.81 (0.63, 1.03)
- Intermediate (neither and more)	204 / 435278	47	Ref.	Ref.	Ref.
- Definite evening	50 / 96943	52	1.10 (0.81, 1.50)	1.08 (0.79, 1.47)	0.95 (0.69, 1.30)
Per 1-level increase toward evening	354 / 801141	44	**1.23 (1.05, 1.45)**	**1.21 (1.03, 1.43)**	1.11 (0.95, 1.31)
P Value for Trend	NA	NA	**0.0107**	**0.0187**	0.1961

* Model 1: Age, ethnic background, census-tract median family income, marital status, workplace, family history of MI and/or stroke, menopausal status, and postmenopausal hormone therapy.

† Model 2: Model 1 + history of diabetes/hypertension/hypercholesterolemia, aspirin use, smoking status, sleep duration, physical activity, body mass index (BMI), Alternate Healthy Eating Index-2010 score (AHEI-2010), and alcohol use. Census-tract median family income, AHEI-2010 score, BMI, physical activity, and alcohol use were modeled as continuous variables.

NA: Not Applicable

medRxiv preprint doi: https://doi.org/10.64898/2026.09.16.26363233; this version posted September 17, 2026. The copyright holder for this preprint (which was not certified by peer review) is the author/funder, who has granted medRxiv a license to display the preprint in perpetuity.

It is made available under a CC-BY 4.0 International license .

Supplemental Table 5. Joint association of shift work and chronotype with risks of cardiovascular disease and all-cause mortality in the NHSII (= 69,458)

	**Duration of rotating night shift work**					
	None		<5 years		≥5 years	
	Association with Incident CVD					
**Chronotype**	Cases / PY; Crude incidence	HR (95% CI)	Cases / PY; Crude incidence	HR (95% CI)	Cases / PY; Crude incidence	HR (95% CI)
Definite morning	90 / 76394; 118	0.95 (0.73, 1.23)	133 / 133720; 99	0.82 (0.64, 1.03)	84 / 58634; 143	1.18 (0.90, 1.5
Intermediate	143 / 118685; 120	Reference	247 / 214447; 115	0.99 (0.81, 1.22)	151 / 101882; 148	1.20 (0.95, 1.5
Definite evening	32 / 22115; 145	1.15 (0.79, 1.70)	79 / 46491; 170	**1.38 (1.05, 1.82)**	46 / 28263; 163	1.29 (0.92, 1.8
	Association with All-Cause Mortality					
Definite morning	116 / 76841; 151	**0.75 (0.60, 0.94)**	272 / 134380; 202	1.03 (0.86, 1.22)	128 / 59100; 217	1.08 (0.87, 1.3
Intermediate	240 / 119454; 201	Reference	457 / 215874; 212	1.08 (0.93, 1.27)	273 / 102641; 266	**1.30 (1.09, 1.5**
Definite evening	50 / 22283; 224	0.97 (0.71, 1.31)	134 / 46890; 286	**1.32 (1.07, 1.64)**	107 / 28581; 374	**1.69 (1.34, 2.1**

Models adjusted for age, ethnic background, census-tract median family income, marital status, workplace, family history of MI and/or stroke menopausal status, and postmenopausal hormone therapy.
HR: Hazard Ratio; PY: Person-Year

medRxiv preprint doi: https://doi.org/10.64898/2026.09.16.26363233; this version posted September 17, 2026. The copyright holder for this preprint (which was not certified by peer review) is the author/funder, who has granted medRxiv a license to display the preprint in perpetuity.

It is made available under a CC-BY 4.0 International license .

Supplemental Figure 1. Study flow diagram

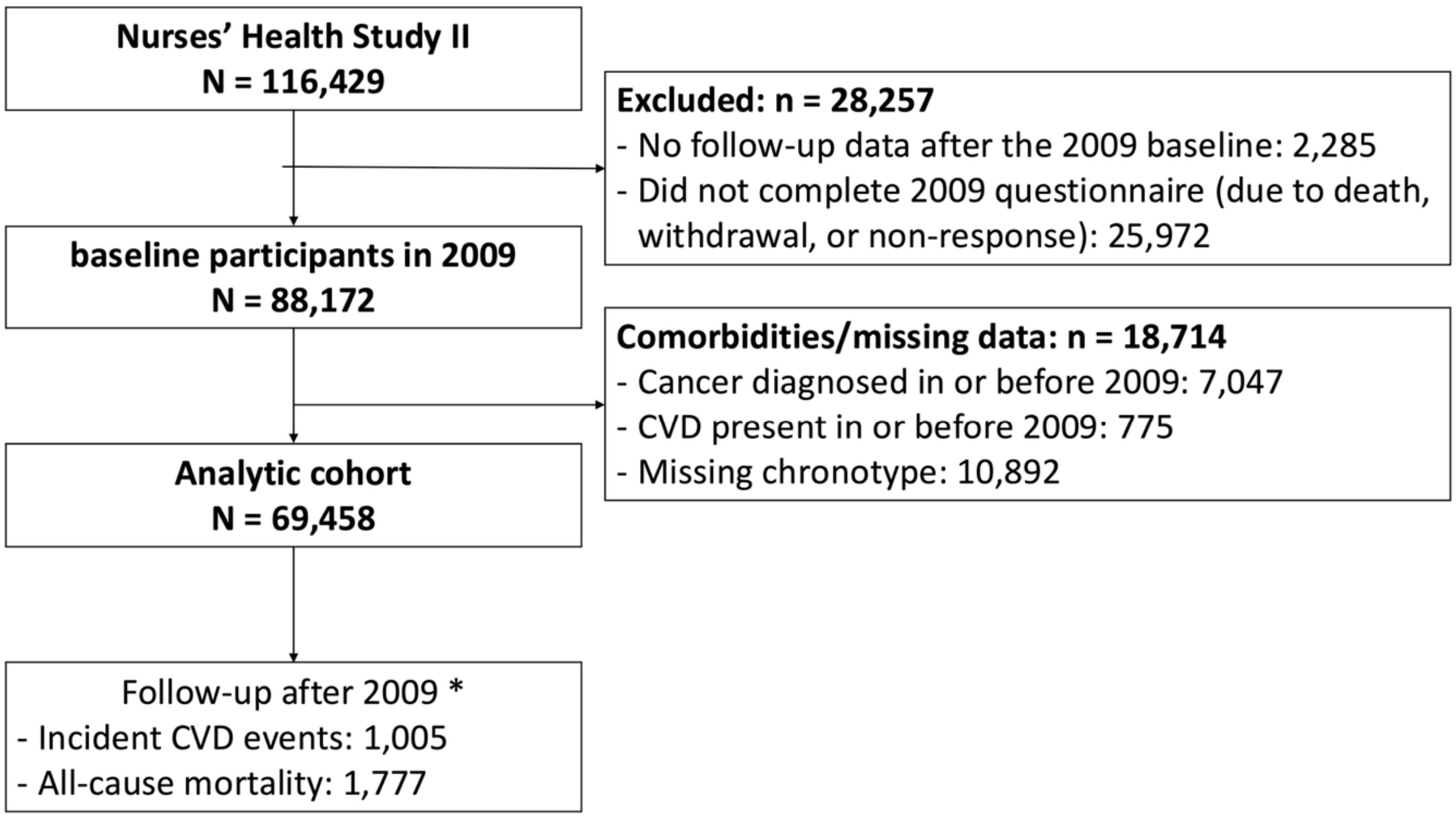

* Follow-up began at the 2009 questionnaire return date and continued through June 2021. For incident CVD analyses, participants were censored at the earliest of first CVD event (fatal or non-fatal myocardial infarction or stroke), death, loss to follow-up, or end of follow-up; for all-cause mortality analyses, participants were censored at death or end of follow-up. Both outcomes were analyzed in separate person-period datasets with time-updated covariates.

medRxiv preprint doi: https://doi.org/10.64898/2026.09.16.26363233; this version posted September 17, 2026. The copyright holder for this preprint (which was not certified by peer review) is the author/funder, who has granted medRxiv a license to display the preprint in perpetuity.

It is made available under a CC-BY 4.0 International license .

Supplemental Figure 2. Data collection timeline for exposures, covariates, and outcome ascertainment

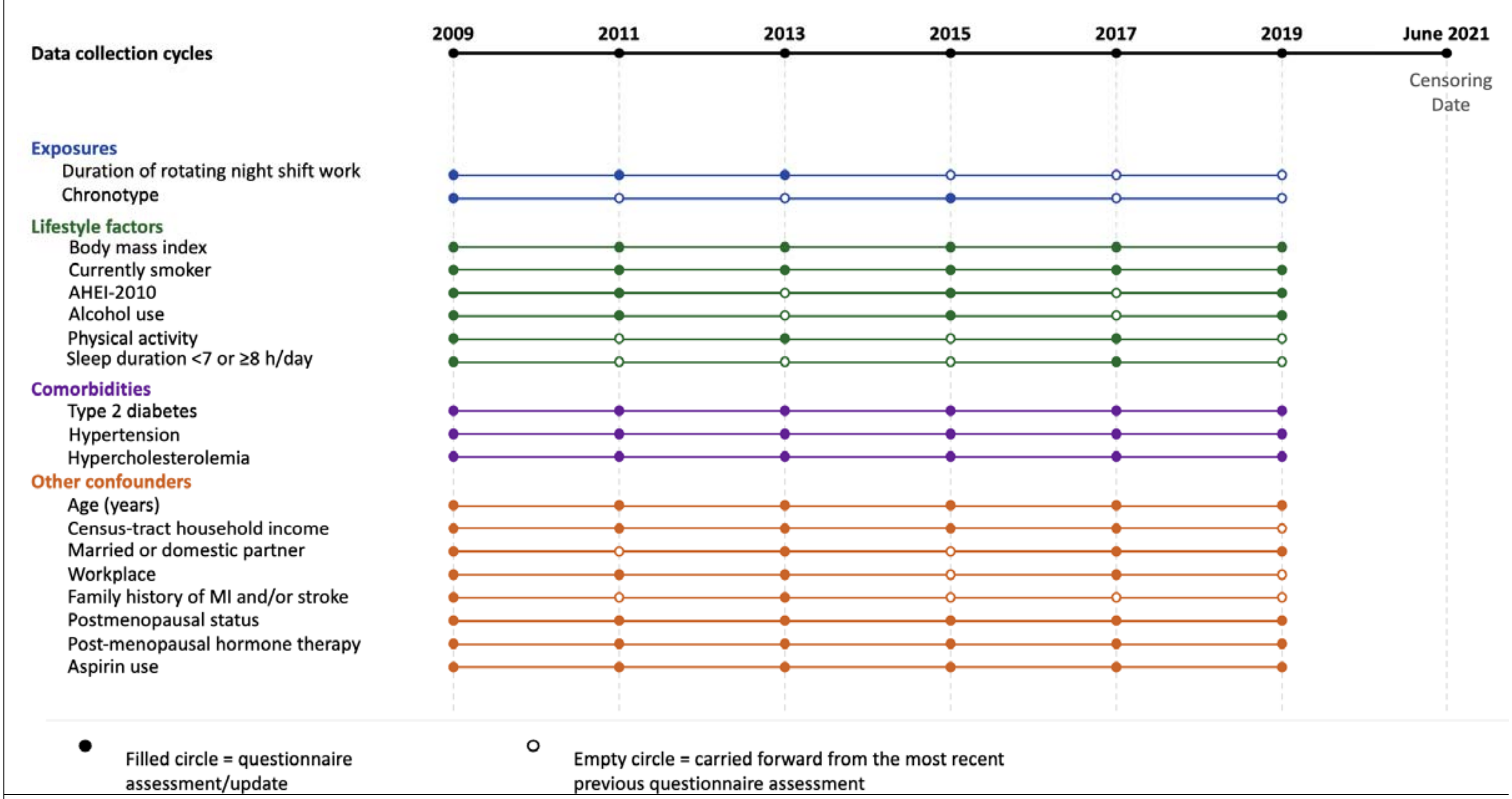

Covariate updating ended in 2019; only outcomes were ascertained after 2019 through June 2021.
